# Effect of pregnancy on bioprosthetic structural valve degeneration

**DOI:** 10.1016/j.ijcchd.2024.100559

**Published:** 2024-12-20

**Authors:** Joshua J. Price, Kalani Ruiz, Jaewon Lim, Yuli Kim, Jonathan Buber

**Affiliations:** a Department of Cardiology, University of Washington, Seattle, WA, USA; b Division of General Internal Medicine, University of Washington, Seattle, WA, USA; c Department of Biostatistics, University of Washington, Seattle, WA, USA; d Division of Cardiology, Hospital of the University of Pennsylvania, Philadelphia, PA, USA

**Keywords:** Cardio-obstetrics, Valve disease, Congenital heart disease

## Abstract

**Background:**

Despite increasing frequency of pregnancies among patients with cardiac conditions, including the presence of prosthetic heart valves, data on the effects of physiological changes during pregnancy on the function of bioprosthetic valves remains scarce and shows conflicting results.

**Objectives:**

This study was aimed to determine the effect of pregnancy on the rate of bioprosthetic structural valve degeneration.

**Methods:**

We designed a retrospective matched cohort study of patients seen between June 2018 and February 2023. All pregnant patients with bioprosthetic valves were matched to non-pregnant controls with prior valve replacement based on bioprosthetic valve location, time since valve implantation, age of the patient at valve implantation and time between baseline and follow up echocardiograms. Echocardiograms of pregnant patients were evaluated for bioprosthetic structural valve degeneration grade based on a dedicated scale before and after pregnancy. Non-pregnant controls had echocardiogram scoring of structural valve degeneration over a similar time period.

**Results:**

Thirty four pregnant patients with bioprosthetic valves in the pulmonary, aortic and mitral positions were matched with 71 non-pregnant controls with identical bioprosthetic valves locations. Over a median follow up period of 13.5 months that included the gestational period, 18 (53 %) pregnant patients had an increase in structural valve degeneration score as compared to 17 (26 %) of the non-pregnant patients in median follow up of 13.7 months (OR 3.87, p = 0.004). On multivariable analysis, pregnancy was the only variable associated with increased structural valve degeneration score.

**Conclusions:**

Our results suggest that pregnancy is associated with increased bioprosthetic structural valve degeneration.

## Highlights

• Bioprosthetic valves degenerate faster during pregnancy compared to non pregnant controls.
• Both left sided and right sided prosthetic valves experienced degeneration during pregnancy.
• Most pregnant women experienced some degree of prosthetic valve degeneration during pregnancy.
• Aspirin is not associated with less valve degeneration during pregnancy.

## Graphical abstract

**Figure:**
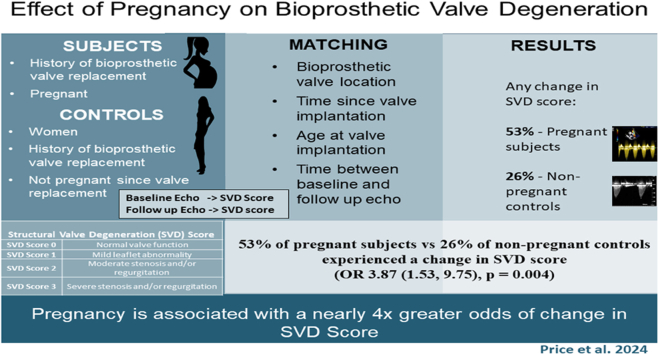

## 1 Introduction

In patients of childbearing age who desire pregnancy and require a heart valve replacement, bioprosthetic valves may be preferred over mechanical valves due to the high rates of maternal and fetal adverse events with systemic anticoagulation [1,2]. With time, bioprosthetic structural valvular degeneration (SVD) occurs in all patients and is related to several processes, including leaflet calcification, “wear and tear” of the valve leaflets, and an immune-mediated response [3]. SVD can lead to valve stenosis, regurgitation, or mixed disease which, if left untreated, may lead to significant morbidity and need for urgent re-intervention. Multiple risk factors for faster SVD have been described, including location of the valve, patient age at the time of implantation, and patient comorbidities including hypertension, type 2 diabetes mellitus, obesity, renal disease and coronary artery disease [4,5].

Pregnancy constitutes a period of both high cardiac output and hypercoagulability, which theoretically may hasten degeneration of bioprosthetic valves [6,7]. Whether the gestational and the early post-partum periods in which these alterations occur are sufficient to cause a more rapid SVD is still debated. Studies thus far have yielded conflicting data on the effects of pregnancy on bioprosthetic valve longevity, with some suggesting pregnancy as a risk factor for faster SVD while others showing no such effect [[8], [9], [10], [11], [12]]. Most of these studies are now several decades old and many involve primarily patients with rheumatic left sided heart disease, which may not reflect more contemporary Cardio-Obstetrics care standards.

With these data and questions in mind, our study aimed to investigate the potential effects of pregnancy on bioprosthetic valve function, and specifically whether gestational state may cause faster bioprosthetic SVD.

## 2 Methods

The study was reviewed and approved by the Institutional Review Board, and no written informed consent was required. A database of all pregnant patients with heart disease who were cared for at our hospital between June 1, 2018, and February 28, 2023, was searched for those with a history of prior bioprosthetic heart valve replacement. Inclusion criteria consisted of history of bioprosthetic valve replacement, age greater than 18, availability of demographic information, a baseline echocardiogram (within 12 months of becoming pregnant through the first 13 weeks or pregnancy) and a follow up echocardiogram performed at least 3 months postpartum, at which time the gestational effects on hemodynamics are mostly resolved [13], but not more than 15 months postpartum at which time regular chronologic changes can affect valve function. Exclusion criteria included mechanical heart valves, pregnancies that ended earlier than 26 weeks of gestation, endocarditis of current bioprosthetic valve, and incomplete data availability for review. All clinical information was extracted from the electronic medical record, and echocardiograms were reviewed in a fashion that was blinded to the original interpretation. For patients who had more than one pregnancy, only the first pregnancy was included in this analysis to avoid the potential effects of multiparity on the valves.

A database of all patients cared for at the adult congenital heart disease clinic between May 1, 2014, and February 28, 2023, was searched for all female patients with history of bioprosthetic heart valve replacement. Patients were eligible to be included as controls if they were women, were older than 18 years, had history of bioprosthetic valve replacement and had at least 2 echocardiograms obtained 6 months apart from each other and available for review. Patients could serve as controls if they had been pregnant prior to the bioprosthetic valve implantation but were excluded if they were pregnant after the valve implantation.

Individual matching of exposed (pregnant after valve implantation) patients to unexposed (not pregnant after valve implantation) patients was completed. Given the small sample size, all potential controls meeting the inclusion criteria were included in matching, leading to multiple unexposed controls being matched to each subject. Unexposed patients were matched to subjects by four criteria: Valve position (pulmonary, aortic, tricuspid or mitral), age at valve implantation, time since valve implantation at time of baseline echocardiogram, and interval between baseline and follow up echocardiograms. During the matching process, controls were forced to match bioprosthetic valve position exactly. To minimize standard error, pregnant patients were assigned to one of 19 strata based on parameters discussed above. A distance formula was used to match control patients with pregnant patients. The matching equation is provided in Supplemental Table 1.

The outcome of this study was defined as a change in the SVD score on follow up echocardiogram compared to baseline echocardiogram. The original SVD score published by Dvir et al. was applied only to bioprosthetic aortic valves [14]. We established an “SVD score” for use in bioprosthetic valves in any position using the same schema (Table 1, Supplemental Table 2). Each echocardiogram was completed at the study center and the SVD score was determined based on the predetermined criteria. Scores were assigned independently by two different authors with expertise in echocardiographic interpretation (JJP and JB) who reviewed the original images. Interpreters were blinded to whether participants were in the pregnancy or control group. Any disagreement was resolved by independent review by a third cardiologist.

**Table 1:** Structural valve disease scores and definitions.

Score	Definition
SVD Score 0	No change from post implantation
SVD Score 1	Mild leaflet abnormality without significant hemodynamic changes
SVD Score 2S	Moderate stenosis
SVD Score 2R	Moderate regurgitation
SVD Score 2RS	Moderate stenosis and regurgitation
SVD Score 3	Severe stenosis and/or severe regurgitation

Table 1. Bioprosthetic valves were assigned an SVD score based on these definitions [14]. Specific criteria for each score can be found in Supplemental Table 1. SVD - structural valve degeneration.

Wilcoxson rank sum test, Fisher's exact test, and Pearson's Chi-squared tests were used to evaluate differences in continuous and categorical variables respectively between subjects and controls at baseline. Unmatched, univariate conditional logistic regression was used to evaluate for change in SVD score by valve location in both exposed and unexposed patients. Variables chosen a priori to be included in a multivariable logistical regression comparing change in SVD score between pregnant patients and matched non pregnant controls included pregnancy, any systemic anticoagulation including heparin or warfarin, presence of any “risk factor” and baseline SVD score. Any “risk factor” was defined as having any of the following: tobacco use, ever having used intravenous drugs, obesity, hypertension, or diabetes. One author (JJP) had full access to all the data in the study and takes responsibility for its integrity and the data analysis.

## 3 Results

Thirty-four pregnant patients with a bioprosthetic valve were identified as subjects and 71 patients were identified to serve as matched controls. Baseline demographics are reported in Table 2. Pregnant patients were numerically but not statistically younger (30.0 vs 32.4 years, p = 0.3) and had more history of ever intravenous drug use (12%vs 4 %, p = 0.03). Baseline SVD score of 0, suggesting normal prosthetic valve function, was more common in controls than pregnant patients while a baseline SVD score of 2, suggesting moderate prosthetic valve dysfunction, was more common in pregnant patients than controls. The majority of the patients were white, average body mass index was mildly elevated and the majority of the patients (65 % in the pregnancy group, 63 % in the matched group) were on aspirin therapy at baseline. The most common underlying cardiac condition in both groups was repaired tetralogy of Fallot followed by bicuspid aortic valve. A complete list of the congenital and acquired heart conditions in the pregnant and the control groups is shown in Supplemental Table 3. The most common bioprosthetic valve position was pulmonary (68 % in controls; 69 % in pregnant subjects) followed by aortic (21 % in controls; 24 % in pregnant subjects) and mitral (12 % in controls; 7 % in pregnant subjects). No subjects, and therefore no controls, had undergone tricuspid valve replacement. The average time from valve implantation to the baseline echocardiogram was 6.5 years in the subjects and 5.5 years in controls (p = 0.3).

**Table 2:** Descriptive statistics of matched exposed and unexposed patients.

Characteristic	Pregnant Subjects (N = 34)	Non-pregnant Controls (N = 71)	p-value
Age at baseline echo (years)	30.0 (±4.7)	32.4 (±7.5)	0.3
Race			0.6
White	31 (91 %)	65 (92 %)	
Black or African American	1 (2.9 %)	3 (4.2 %)	
American Indian or Native Alaskan	0 (0 %)	2 (2.8 %)	
Asian	1 (2.9 %)	1 (1.4 %)	
Native Hawaiian or other Pacific Islander	1 (2.9 %)	0 (0 %)	
BMI	27.42 (±6.1)	28.74 (±7.9)	0.8
Lesion			0.4
Tetralogy of Fallot	17 (50 %)	33 (46 %)	
Bicuspid aortic valve	7 (21 %)	16 (23 %)	
AVSD	0 (0 %)	2 (2.8 %)	
Ross operation	0 (0 %)	6 (8.5 %)	
Others	10 (29 %)	15 (21 %)	
Systemic right ventricle	1 (2.9 %)	2 (2.8 %)	>0.9
Hypertension	2 (5.9 %)	6 (8.5 %)	>0.9
Diabetes	1 (2.9 %)	2 (2.8 %)	>0.9
Obesity	8 (24 %)	13 (18 %)	0.5
Tobacco	5 (15 %)	4 (5.6 %)	0.15
Intravenous drug use	4 (12 %)	1 (1.4 %)	0.037
Aspirin use	22 (65 %)	45 (63 %)	0.9
Beta blocker use	12 (35 %)	23 (32 %)	0.8
Systemic anticoagulation	1 (2.9 %)	6 (8.5 %)	0.4
Systemic left ventricular ejection fraction	63.3 (±6.1)	60.9 (±6.8)	0.3
Moderate or worse systemic right ventricular dysfunction	0 (0 %)	1 (1.4 %)	>0.9
Moderate or worse subpulmonary ventricular dysfunction	0 (0 %)	5 (7 %)	0.2
Valve age at baseline (years)	6.5 (±5.2)	5.5 (±5.2)	0.3
Valve location			0.7
Pulmonary	23 (68 %)	49 (69 %)	
Melody valve in the pulmonary position	5 (15 %)	6 (9 %)	0.3
Aortic	7 (21 %)	17 (24 %)	
Mitral	4 (12 %)	5 (7 %)	
SVD score at baseline			0.005
0	8 (24 %)	37 (52 %)	
1	10 (29 %)	15 (21 %)	
2R	5 (15 %)	6 (8.5 %)	
2S	2 (5.9 %)	0 (0 %)	
2RS	9 (26 %)	8 (11 %)	
3	0 (0 %)	5 (7.0 %)	
Valve replacement during follow up	15 (44 %)	12 (17 %)	0.003
Death	0	1 (1.4 %)	>0.9

Table 2. Baseline data for pregnant subjects and not pregnant controls. Continuous data: Mean (SD); Categorical data: n (%). BMI - body mass index; AVSD - atrio-ventricular septal defect. Other abbreviations as in previous tables.

Pregnancy data is presented in Table 3. All pregnancies resulted in live births and there were no miscarriages. The average age at time of conception was 30.1 years and the average gestational age at the time of delivery was 37.8 weeks. Six percent of pregnancies were complicated by pre-eclampsia. Sixty two percent of patients were on aspirin therapy throughout pregnancy (one stopped the treatment due to epistaxis) and 41 % were on beta blocker therapy.

**Table 3:** Descriptive statistics among pregnant patients.

Characteristic	Pregnant Subjects (N = 34)
Age at pregnancy (years)	30.1 (±5.5)
Valve age at baseline (years)	6.5 (±5.2)
Gestational age at delivery (weeks)	37.8 (±2.7)
Twin gestation	1 (2.9 %)
Pregnancy complications	
Preeclampsia	2 (5.9 %)
Gestational diabetes	1 (2.9 %)
Gestational hypertension	0 (0 %)
Pregnancy Medications	
Aspirin	21 (62 %)
Beta blocker	14 (41 %)
Coumadin	1 (2.9 %)
Heparin before third trimester	1 (2.9 %)

During a mean follow up duration of 13.5 months from the time of the baseline pre-pregnancy echocardiogram to the postpartum echocardiogram, 18 patients (53 %) of the pregnant cohort had an increase in the SVD score, whereas the corresponding rates among the non-pregnancy matched group were 26 % (17 patients) during a similar follow up duration of 13.7 months (Odds Ratio [OR] 3.87, 95 % Confidence Interval [CI] 1.53, 9.75; p = 0.004). (Graphical abstractFig. 1, Supplemental Table 4). In a multivariable regression analysis that included gestational state, antiaggregant/anticoagulation therapy, presence of one or more SVD risk factors (tobacco use, intravenous drug use, obesity, hypertension, or diabetes) and baseline SVD score, only pregnancy was associated with an increase in the SVD score (OR 3.81, 95 % CI 1.3, 10.8; p = 0.004). (Table 4a, Table 4b, Table 4c). None of the patients underwent a valve replacement operation (surgery or trans-catheter) during the follow up period.

**Fig. 1:**
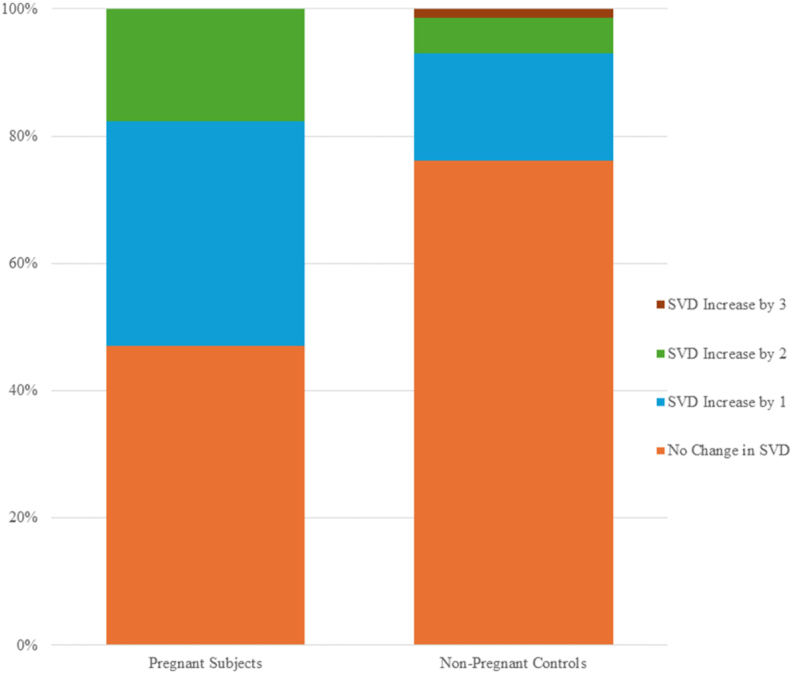
Change in SVD score between subjects and controls. Percentage of patients experiencing a change in SVD score between baseline and follow up echocardiogram. 53 % of pregnant subjects vs 26 % of non-pregnant controls experienced a change in SVD score (OR 3.87 (1.53, 9.75), p = 0.004). SVD – structural valve degenderation.

**Table 4a:** Univariate conditional logistic regression analysis of matched data.

Variable	OR (95 % CI)
Pregnancy	3.87 (1.53, 9.75) p = 0.004

**Table 4b:** Multivariable conditional logistic regression analysis of matched data.

Variable	OR (95 % CI)
Pregnancy	3.93 (1.54, 9.99) p = 0.004
Antiaggregant/Anticoagulation therapy	0.85 (0.31, 2.30)
Any risk factor^1^	1.06 (0.42, 2.69)

1 Risk factors included: tobacco use, intravenous drug use, obesity, hypertension, or diabetes.

**Table 4c:** Multivariable conditional logistic regression analysis of matched data.

Variable	OR (95 % CI)
Pregnancy	3.81 (1.3, 10.8) p = 0.004
Antiaggregant/Anticoagulation therapy	0.94 (0.31, 2.75)
Any risk factor	0.98 (0.36, 2.65)
Baseline SVD = 0	Reference
Baseline SVD = 1	0.45 (0.10, 1.90)
Baseline SVD = 2	1.05 (0.32, 3.43)

In a separate, non-matched analysis, no differences were observed in frequency of SVD based on the valve position. This was consistent when analysis was done on pregnant patients, non-pregnant patients, or the entire combined cohort.

## 4 Discussion

The results of our study suggest that the physiological changes that occur during pregnancy and that include high output state, hypercoagulability and a change in the hormonal profile among others, may lead to a more rapid bioprosthetic valve degeneration. Our analysis shows that pregnancy is associated with a nearly 4-fold increase in the risk for faster structural valve degeneration in the pulmonary, aortic and mitral positions. While this was a single center analysis and its findings need to be validated in larger cohorts, the meticulous matching process and close follow up imaging studies confer a clinical relevance which may affect pre-conception counselling and follow up protocols during and after pregnancy.

Previous studies that evaluated the effects of pregnancy on bioprosthetic valve function showed conflicting results. In an analysis of 87 patients with a porcine or pericardial valve, Badduke et al. found that pregnancy was associated with structural valve degeneration [8]: of the 17 patients who became pregnant, SVD occurred in 47.1 %, vs. 14.3 % of the nonpregnancy group (p < 0.05). Reoperation was performed in 59 % of the pregnancy group and 19 % of the nonpregnancy group, primarily for degeneration manifested as valvular obstruction from aggressive calcification.

Conversely, El Shaer et al. examined 85 female patients who underwent bioprosthetic valve replacement and found that those who had become pregnant had similar rates of valve replacement compared to those who had not become pregnant [9]. They concluded that pregnancy was not a risk factor for bioprosthetic SVD. Similarly, a prospective study by Avila et al. failed to find increased SVD in 48 pregnant patients compared to 37 non-pregnant patients after 5 years of follow up [10].Finally, a study by North et al. showed no increased risk of bioprosthetic valve degeneration or valve loss in retrospective review of 78 women with bioprosthetic valves [2].

While the above-mentioned studies primarily addressed SVD in left sided heart valves (mitral in older cohorts, aortic in more recent studies), our more contemporary cohort included pregnant patients and matched non-pregnant female controls with a variety of congenital and acquired valve disease. The majority of our subjects had congenital heart disease with nearly half having bioprosthetic valve in the pulmonary position. We were able to clearly demonstrate baseline and follow up valve function and included baseline SVD scores in our multivariable model.

Our study is unique and important in several regards. First, we used matching to identify non pregnant controls that closely resembled the pregnant subjects. Compared to pregnant patients, controls had identical positions of their bioprosthetic valves, were similar in age at the time of valve replacement, similar in age at the time of baseline echocardiogram and had a similar time between baseline and follow up echocardiograms. This is important to remove potential known confounders that can lead to different rates of SVD. We do note that the valves in the pregnant patients were about 1 year older than the matched controls. While this was statistically not significant it may play a role clinically though likely relatively minor. Second, our study uses precise criteria to look for small changes in valve function. Prior studies used macro endpoints including need for valve replacement and mortality which are important clinical end points, but also subject to confounding. By looking at changes in valve function we are potentially able to identify changes sooner and in a more precise way. Finally, we intentionally chose a small time period centered around pregnancy in order to isolate the effects of pregnancy as much as possible. By choosing a follow up time that is only a few months after the completion of pregnancy we remove potential competing factors for SVD. This is an important first step and we acknowledge the need for further follow up to consider long term implications of pregnancy on bioprosthetic valves as well as impact of multiple pregnancies on a single valve.

We hypothesize that the increased change in SVD score seen in our study was related to the physiologic changes associated with normal pregnancy as discussed above, all of which may lead to hastened “wear and tear” phenomenon of the prosthetic valve tissue. Another theory is thatincreased small thrombus formation on the valve leaflets due to the hypercoagulable state induced by pregnancy leads to progressive degeneration. About two thirds of both pregnant and non-pregnant controls were on aspirin with very few on any systemic anticoagulation suggesting that if hypercoagulability does play a role in SVD, aspirin may be insufficient to prevent it from having a clinical effect. While our study is underpowered to examine the impact of pre-eclampsia on SVD progression as only 2 of our pregnant patients developed it during their pregnancy, pre-eclampsia is a multi-systemic disease that may worsen SVD progression through the wear and tear, worse cardiac function and hypercoagulable state mechanisms and future larger studies that will examine these effects are warranted. Further work will also be needed to determine which factors are most responsible for SVD in this patient population and what, if any, interventions can be undertaken to prevent degeneration.

Valve degeneration did not differ based on location in our study, finding that was true for both the pregnant and the matched groups. Because valve location was included in our matching scheme this was necessarily an unmatched, univariate analysis that may be subject to confounding, especially considering prior literature suggesting that valve location is an important predictor of valve longevity [15]. This deserves further evaluation in the future as differences could impact patients’ decisions on when to undergo valve replacement relative to becoming pregnant.

Pregnant subjects had higher SVD score compared to the non-pregnant controls; only 24 % of pregnant subjects had normal bioprosthetic valve function (SVD score 0) as compared to 52 % of non-pregnant controls. Conversely, 47 % of pregnant subjects had moderate valve degeneration (SVD score 2) compared to only 20 % of the controls. This is especially notable given the effort to control for known valve degeneration risk factors like valve age at enrollment. One hypothesis, though unexplored, is that women with significant valve degeneration may choose to become pregnant prior to an impending valve replacement and the implications of a procedure for the following 6–12 months. It is important to note our analysis accounts for this difference as the dependent variable in our analysis was defined as any change in SVD score, which is not affected by baseline valve function. By looking for change in valve function, rather than a prespecified end point such as an SVD score of 3, we limit the impact of confounding. Here too, further work with a larger sample size to reduce the random chance of difference in baseline valve is warranted and hopefully will further align with our findings. It will be interesting to learn if this pattern of patients who become pregnant have more rapid valve degeneration compared to their peers who do not become pregnant.

Our work does have several limitations. Its retrospective design has inherent limitations including the inability to draw any conclusions about causation. This is also a small, single center study and may not be generalizable to other patient populations. We acknowledge that our study design includes baseline echocardiograms that may have been done during the first trimester of pregnancy and that follow up echocardiograms could be done as early as 3 months postpartum. We chose these parameters intentionally to capture as many pregnant subjects as possible with the trade-off of potential confounding. However, we feel that the hemodynamic changes in the first 13 weeks of pregnancy are usually quite minimal and should have little clinical impact on their echocardiograms. And while the hemodynamic effects of pregnancy may not have completely normalized until 6 months postpartum, we wanted to capture data points from women seen 3–6 months postpartum for a follow up visit. We limited confounding from residual increased cardiac output and blood volume by calculating doppler velocity indices to not over diagnose worsening valve stenosis. Finally, it is also important to note that a single increase in SVD stage may not be a clinically relevant change. Despite these limitations this work is an important addition to the literature addressing an unanswered question that is frequently considered in cardio-obstetrical care.

## 5 Conclusion

In conclusion, our matched cohort study suggests that pregnancy is associated with a nearly 4-fold increase in the risk of structural bioprosthetic valve degeneration compared to non-pregnant patients. This risk of faster SVD does not appear to be explained by baseline valve function. The risk of valve degeneration may be an important factor in patients’ decision to become pregnant, though further work is needed to corroborate our findings before firm recommendations can be made.

## CRediT authorship contribution statement

**Joshua J. Price:** Writing – review & editing, Writing – original draft, Validation, Project administration, Methodology, Investigation, Formal analysis, Data curation. **Kalani Ruiz:** Writing – review & editing, Writing – original draft, Methodology, Formal analysis, Data curation. **Jaewon Lim:** Writing – review & editing, Writing – original draft, Formal analysis. **Yuli Kim:** Writing – review & editing, Methodology, Investigation, Formal analysis, Conceptualization. **Jonathan Buber:** Writing – review & editing, Writing – original draft, Validation, Supervision, Resources, Methodology, Investigation, Formal analysis, Data curation, Conceptualization.

## Author disclosures

None. There are no relationships with industry.

## Declaration of competing interest

The authors declare that they have no known competing financial interests or personal relationships that could have appeared to influence the work reported in this paper.
