# Supplementary material for: Effect of pregnancy on bioprosthetic structural valve degeneration
Source: Int J Cardiol Congenit Heart Dis. 2024 Dec 20;19:100559. doi: 10.1016/j.ijcchd.2024.100559 (PMC11803119; doi:10.1016/j.ijcchd.2024.100559)
Supplement: Multimedia component 1 [file mmc1.docx]

**Supplemental Table 1**. Matching Formula

Matching formula:

Distance = I(VS_ex_≠VS_unex_)∗9999+ |VT_ex_−VT_unex_|+|VA_ex_−VA_unex_|+|IT_ex_−IT_unex_|

VS - Valve site; VT - time since last valve replacement; VA - Age at valve replacement; IT - Interval between echocardiograms; ex - exposed; unex - unexposed

**Supplemental Table 2.** SVD score grading criteria.

- Mitral valve
- Stenosis:
- 2.0 - 2.5 cm2; increased inflow velocities - Mild
- 1.6-2.0 cm2 - Moderate
- < or = 1.5 cm2 – Severe
- Regurgitation:
- EROA <0.20; VC <0.3 - mild
- 0.20-0.39; VC 0.3-0.7 - moderate
- 0.40; VC >0.7 - severe
- Aortic valve
- Stenosis
- Vmax 2-2.9 - mild
- Vmax 3-3.9 - moderate
- Vmax >4, AVA <1.0- severe
- Regurgitation
- EROA < 0.1, VC < 0.3, deceleration half time >500ms - mild
- EROA 0.1-0.3, VC 0.3-0.6, deceleration half time 500-200ms – moderate
- EROA >0.3, VC >0.6, deceleration half time <200ms – severe
- Pulmonary valve
- Stenosis
- Vmax 2-2.9 - mild
- Vmax 3-3.9 - moderate
- Vmax >4
- Regurgitation
- Thin jet, <10mm in length – mild
- Not mild, not severe – moderate
- PR half time <100, PR jet width/PV annulus >0.7, reversal in PAs – severe

**Supplemental Table 3.** Heart disease in pregnant and non-pregnant patients

| Heart disease | Pregnant Subjects N=34 | Non-Pregnant Controls N=71 |
| --- | --- | --- |
| Tetralogy of Fallot | 17 (50%) | 33 (46%) |
| Bicuspid aortic valve | 7 (21%) | 16 (23%) |
| AVSD | 0 (0%) | 2 (3%) |
| Ross | 0 (0%) | 6 (9%) |
| L-TGA | 1 (3%) | 2 (3%) |
| Rheumatic heart disease | 2 (6%) | 1 (1%) |
| ASD | 2 (6%) | 0 (0%) |
| VSD | 1 (3%) | 0 (0%) |
| DORV | 1 (3%) | 0 (0%) |
| Endocarditis | 1 (3%) | 0 (0%) |
| Truncus arteriosus | 2 (6%) | 1 (1%) |
| D-TGA, atrial switch | 0 (0%) | 3 (4%) |
| Congenital PS | 0 (0%) | 3 (4%) |
| PA/IVS | 0(0%) | 3 (4%) |
| NICM | 0 (0%) | 1 (1%) |

Complete list of heart disease in both pregnant and non-pregnant patients. AVSD – atrioventricular septal defect; L-TGA – L-transposed great arteries; ASD – atrial septal defect; VSD – ventricular septal defect; DORV – double outlet right ventricle; D-TGA – D-transposed great arteries; PS – pulmonary stenosis; PA/IVS – pulmonary atresia with intact ventricular septum; NICM – non ischemic cardiomyopathy.

**Supplemental Table 4.** Follow up SVD sore by baseline SVD score in pregnant subjects and non-pregnant controls.

Pregnant Subjects

|  | | SVD at follow up | | | | | | |
| --- | --- | --- | --- | --- | --- | --- | --- | --- |
|  |  | Stage 0 | Stage 1 | Stage 2S | Stage 2R | Stage 2RS | Stage 3 | Total (%) |
| SVD at baseline | Stage 0 | 3 | 4 | 1 | 0 | 0 | 0 | 8 (24%) |
|  | Stage 1 | 0 | 6 | 1 | 3 | 0 | 0 | 10 (29%) |
|  | Stage 2S | 0 | 0 | 5 | 0 | 1 | 3 | 9 (26%) |
|  | Stage 2R | 0 | 0 | 0 | 2 | 1 | 2 | 5 (15%) |
|  | Stage 2RS | 0 | 0 | 0 | 0 | 0 | 2 | 2 (6%) |
|  | Stage 3 | 0 | 0 | 0 | 0 | 0 | 0 | 0 |

Non-pregnant controls

|  | | SVD at follow up | | | | | | |
| --- | --- | --- | --- | --- | --- | --- | --- | --- |
|  |  | Stage 0 | Stage 1 | Stage 2S | Stage 2R | Stage 2RS | Stage 3 | Total  (%) |
| SVD at baseline | Stage 0 | 27 | 7 | 0 | 2 | 0 | 1 | 37 (52%) |
|  | Stage 1 | 0 | 12 | 1 | 2 | 0 | 0 | 15 (21%) |
|  | Stage 2S | 0 | 0 | 6 | 0 | 1 | 1 | 8 (11%) |
|  | Stage 2R | 0 | 0 | 0 | 4 | 1 | 1 | 6 (8%) |
|  | Stage 2RS | 0 | 0 | 0 | 0 | 0 | 0 | 0 |
|  | Stage 3 | 0 | 0 | 0 | 0 | 0 | 5 | 5 (7%) |
